# β3-Adrenoreceptor Blockade Induces Stem Cells Differentiation in Melanoma Microenvironment

**DOI:** 10.3390/ijms21041420

**Published:** 2020-02-20

**Authors:** Maura Calvani, Gennaro Bruno, Annalisa Dabraio, Angela Subbiani, Francesca Bianchini, Filippo Fontani, Gabriella Casazza, Marina Vignoli, Francesco De Logu, Stefano Frenos, Luca Filippi, Claudio Favre

**Affiliations:** 1 Division of Pediatric Oncology/Hematology, Meyer University Children’s Hospital, 50139 Florence, Italy; gennaro.bruno@unifi.it (G.B.); annalisa.d.92@gmail.com (A.D.); angela.subbiani@gmail.com (A.S.); filippo.fontani@unifi.it (F.F.); marina.vignoli@unifi.it (M.V.); 2 Department of Health Sciences, University of Florence, 50139 Florence, Italy; francesco.delogu@unifi.it; 3 Department of Experimental and Clinical Biomedical Sciences, University of Florence, 50139 Florence, Italy; francesca.bianchini@unifi.it; 4 Paediatric Hematology Oncology, Bone Marrow Transplant, S. Chiara University Hospital of Pisa, 56126 Pisa, Italy; g.casazza@ao-pisa.toscana.it; 5 Hematology-Oncology Department, “Anna Meyer Children’s Hospital”, 50139 Florence, Italy; stefano.frenos@meyer.it (S.F.); claudio.favre@meyer.it (C.F.); 6 Neonatal Intensive Care Unit, Medical Surgical Fetal-Neonatal Department, Meyer “University Children’s Hospital, 50139 Florence, Italy; luca.filippi@meyer.it

**Keywords:** β3-adrenoreceptor, differentiation, tumor microenvironment

## Abstract

Although there is an increasing evidence that cancer stem cell (CSC) niches in the tumor microenvironment (TME) plays a crucial role in sustaining solid tumors progression, several molecular players involved in this regulation still remain unknown. The role of β-adrenergic signaling in enhancing tumor growth through β2-adrenoreceptors (β2-ARs) has been confirmed in different cancer models, but the role played by the β3-adrenergic receptor (β3-AR) has recently emerged. Previous studies showed that β3-AR promotes cancer growth through the activation of different stromal cells in the TME, and leads to melanoma malignancy progression through inflammation, angiogenesis, and immunotolerance. Here we show that in B16 melanoma-bearing mice, the pharmacological β3-AR blockade is able to reduce the expression of CSC markers, and to induce a differentiated phenotype of hematopoietic subpopulations in TME. In particular, cytofluorimetric analysis (FACS) of the tumor mass shows that β3-AR antagonist SR59230A promotes hematopoietic differentiation as indicated by increased ratios of lymphoid/hematopoietic stem cells (HSCs) and of myeloid progenitor cells/HSCs, and increases the number of Ter119 and natural killer (NK) precursor cells, and of granulocyte precursors, indicating active hematopoiesis within the tumor tissue. Moreover, pharmacological antagonism of β3-AR induces mesenchymal stem cell (MSC) differentiation into adipocytes subtracting a potential renewal of the stem compartment by these cells. Here we demonstrate that β3-AR blockade in the TME by inducing the differentiation of different stromal cells at the expense of stemness traits could possibly have a favorable effect on the control of melanoma progression.

## 1. Introduction

Tumor microenvironment (TME) represents an organ-like structure that sustains tumor progression. TME contains different types of cells with distinct phenotypic states, including cancer stem cells (CSCs) [1]. CSCs reside in TME stem cell niches that are composed of different stromal cells [2]: fibroblasts, endothelial cells, and the cells recruited from bone marrow (BM) via the systemic circulation (mesenchymal and hematopoietic stem cells), each of which contributes to proliferation, metastasis, and formation of pre-metastatic niches for cancer growth [3,4]. Mesenchymal stem cells (MSCs) provide an advantageous TME for the restoration of CSCs through their ability to home and engraft to solid tumors [5]. Moreover, MSCs are multipotent stem cells that can differentiate into connective tissue cells, such as adipocytes, chondrocytes, and osteocytes [6].

The differentiation grade of tumors is a central aspect in the biology of malignancies. In particular, melanoma shows high cellular heterogeneity, which reflects the different cellular fates of the stem cells during the tumor progression. The differentiation stage is often strongly associated with tumor behavior: undifferentiated tumors are generally more aggressive than the differentiated ones [7]. The differentiation mechanisms of tumor and stem cells within the TME are poorly known, but they have recently been attracting attention as new therapeutic strategies [8].

Recent studies have demonstrated that β-adrenergic signaling enhances tumor growth through β2-adrenoreceptors (β2-ARs), and that propranolol administration (β1- and β2-AR antagonist) provides protection against different types of cancer [9,10,11]. β3-ARs upregulation in the TME promotes cancer growth through the activation of different stromal cells like BM-derived precursors of MSCs and endothelial precursor cells (EPCs) sustaining tumor inflammation and angiogenesis, and promoting melanoma malignancy by achieving stem cell traits [12]. This work reports a new role of β3-ARs in the differentiation of the CSC compartment in the TME, supporting the hypothesis that differentiation could be a valid strategy to counteract melanoma aggressivity.

## 2. Results

### 2.1. β-ARs Blockade Leads to Tumor Growth Reduction by Affecting Proliferation and Viability of Melanoma Cells

**Figure 1:**
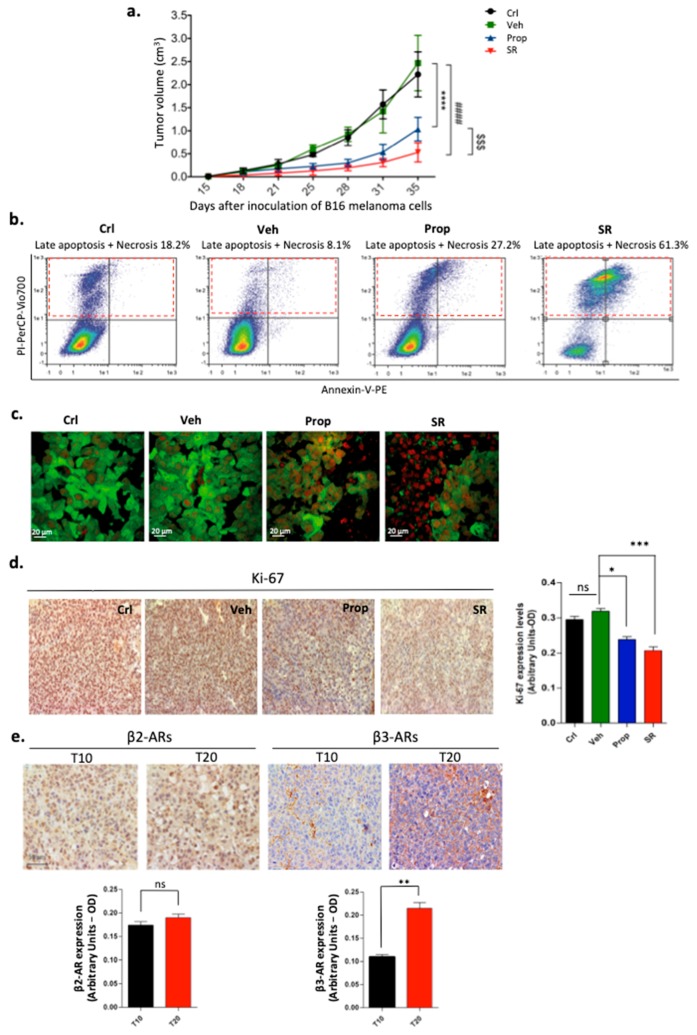
(**a**) Tumor growth rate after the subcutaneous injection of B16-F10 cells in control (Crl), vehicle-(Veh), propranolol-(Prop), or SR59230A-(SR) treated C57BL/6 mice (n = 6). Values are presented as mean ± SD. (**b**) Representative Annexin V/PI staining plots of homogenized tumor samples after 20 days of treatment (T20). (**c**) Confocal microscopy images of PI staining of tumors obtained from mice injected with B16-F10-GFP. (**d**) Representative immunohistochemical staining of proliferation marker Ki-67 in tumor tissues after 20 days of treatment (T20), and relative quantification. (**e**) Representative immunohistochemical staining of β2- and β3-ARs in tumor tissues, and relative quantification after 10 days (T10) and 20 days (T20) of treatment. ns= not significant; β2-Ars = β2-Adrenoreceptors; β3-ARs= β3-Adrenoreceptors.

*In vivo* administration of selective β3-AR antagonist SR59230A markedly reduced tumor growth and weight compared with control and vehicle-treated mice. In contrast, propranolol (a non-selective β1/β2-AR blocker) exerted a lower effect on tumor mass reduction (Figure 1a). The data revealed that, in mice inoculated with B16-F10 cells expressing a green fluorescent protein, the SR59230A-dependent tumor growth reduction was associated with extensive cell death in the tumor mass as indicated by Annexin V/PI assay (Figure 1b). Images of PI staining of tumor sections confirmed the transition from an apoptotic to a massive necrotic phenotype after 20 days of treatment in the tumor mass of SR59230A-treated mice (Figure 1c). Adverse events for the health of propranolol- and SR59230A-treated mice were excluded by performing blood test analysis and by measuring total weight of the mice after 20 days of treatment (Supplementary Figure S2a,b).

Histological analysis after 20 days of treatments with both β-AR antagonists revealed reduced expression of proliferation marker Ki-67 with a more pronounced effect in the SR59230A-treated group (Figure 1d). Immunohistochemistry analysis of the excised tumor mass showed that tumor growth was associated with a progressive increase in β3-AR-enriched cells, but not in β2-ARs (Figure 1e). Literature has previously reported that β3-AR antagonism leads to a decrease in tumor mass *in vivo*, both in melanoma [12,13,14,15,16] and prostate cancer [17]. In fact, β3-AR is correlated with melanoma progression and its expression is increased in melanoma stem cells [12]. Moreover, other studies demonstrated that selective β3-AR antagonism with SR59230A impaired the ability of A375 cells to form melanospheres, and decreased the expression of stem cell markers CD133 and SOX2 [12].

### 2.2. β-ARs Blockade Attenuates the Expression of Stemness Markers

**Figure 2:**
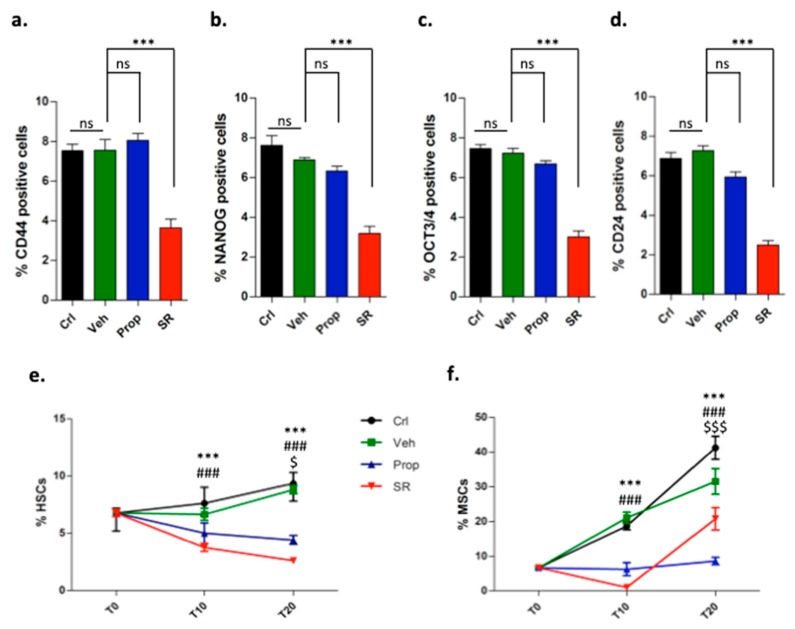
FACS analysis and relative quantification of: (**a**–**d**) CD44^+^, NANOG^+^, OCT3/4^+^, and CD24^+^ cells gated on CD45^−^ tumor cells at T20. (**e**) Hematopoietic stem cells (HSCs) (% of CD117^+^, Sca1^+^ gated on Lin^−^) at the baseline (T0), T10, T20. (**f**) Mesenchymal stem cells (MSCs) (% of CD29^+^, CD44^+^ gated on CD106^+^, Sca1^+^, CD73^+^, CD45^−^, CD11b^−^) at T0, T10, T20.

Results revealed that the expression of stem cell markers identified as CD44, NANOG, OCT3/4, and CD24 in tumor cells was strongly attenuated by SR59230A treatment (Figure 2a–d).

TME stem cell compartment is characterized by high presence of hematopoietic stem cells (HSCs) responsible for poor prognosis [18], and by MSCs, which differentiate mainly toward an immune tolerant phenotype subpopulation of cells, defined as myeloid-derived suppressor cell (MDSC) [16]. Notably, β-AR blockers decreased the percentage of HSCs and MSCs in tumor sites with a stronger activity of SR59230A, demonstrating a direct involvement of β3-ARs in maintaining stemness in the TME (Figure 2e,f).

### 2.3. β3-AR Blockade Induces Tumor Stromal Cells Differentiation

**Figure 3:**
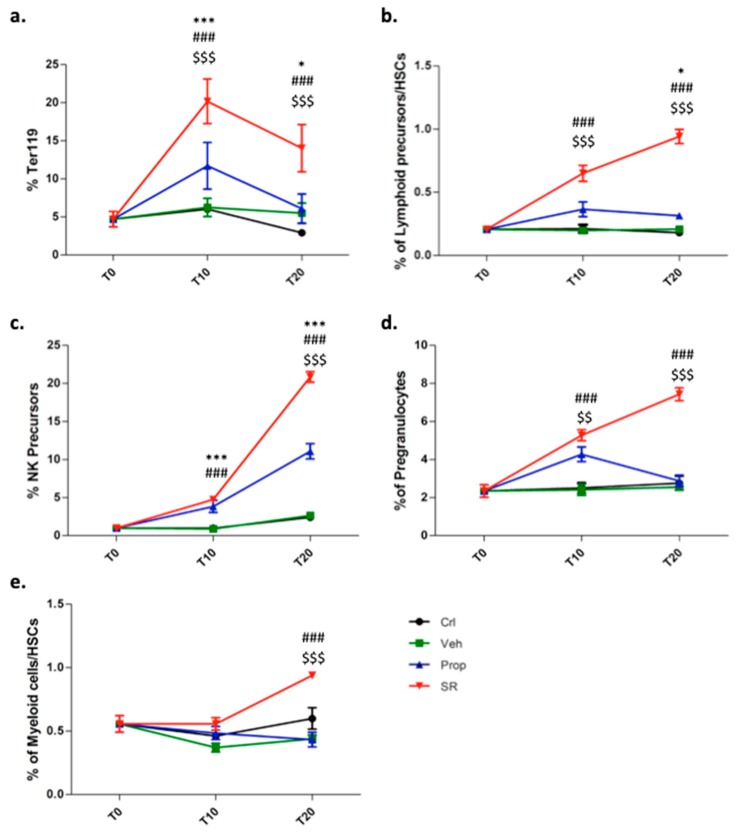
FACS analysis and relative quantification of: (**a**) Ter119 gated on CD45^-^ cells at T0, T10, T20. (**b**) Lymphoid precursors (SCA1^+^ gated on CD117^+^, CD127^+^, Lin^−^)/HSCs ratio at T0, T10, T20. (**c**) Natural killer (NK) precursors (NK1.1^−^ gated on CD122^+^, Lin^−^) at T0, T10, T20. (**d**) Pre-granulocytes (CD127^−^, CD16/32^+^ gated on CD117^+^, SCA1^−^, CD34^+^, Lin^−^) at T0, T10, T20. (**e**) Myeloid (SCA1^−^ gated on CD117^−^, CD127^+^, Lin^−^)/HSCs ratio at T0, T10, T20.

Interestingly, the tumor mass of SR59230A-treated mice exhibited a remarkable increase in the number of cells expressing the erythroblast specific marker, Ter119, used to exclude the presence of erythrocytes during adipocyte detection [19] (Figure 3a). Analysis of the lineage of different hematopoietic cells showed that SR59230A promoted differentiation as indicated by increased ratios of lymphoid/HSCs and myeloid progenitor cells/HSCs (Figure 3b,e). β3-ARs blockade increased the number of Ter119, natural killer (NK) precursor cells, and of granulocyte precursors (Figure 3a,c,d) indicating active hematopoiesis within the tumor tissue.

To assess whether the differentiated cells may be recruited directly from bone marrow, or the differentiation process occurs only in the TME, we analyzed bone marrow of the treated mice. The results obtained demonstrated that differentiation of hematopoietic progenitors occurs in the bone marrow niche (Supplementary Figure S1a) of SR59230A compared to the vehicle-treated mice. Nevertheless, cells derived from the untreated tumor mass, cultured, and treated *ex vivo* with SR59230A, confirmed activation of the differentiation process in the TME of the β3-AR antagonist treated mice (Supplementary Figure S1b).

### 2.4. β-AR Antagonism Increases Pre-Adipocytes Formation in the Tumor Microenvironment

**Figure 4:**
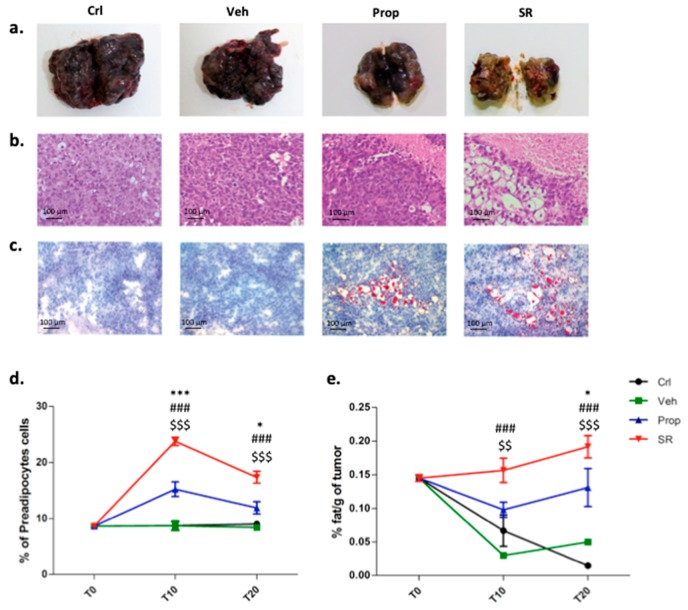
*(***a**) Representative images of excised tumors of the Crl, Veh-, Prop- and SR-treated mice (n = 6) at T20. (**b**) Representative hematoxylin and eosin (H&E) images of a tumor section. (**c**) Histological staining with Oil Red O of tumor sections. (**d**) FACS quantification of pre-adipocytes (CD31^−^, CD34^−^, Sca1^+^, Ter119^−^) at T0, T10, T20. (**e**) Fat measurement (lipid/g of tumor) using the Folch method at T0, T10, T20.

Excised SR59230A-treated masses appeared capsulated, yellowish, and greasy to the touch, similar to fat tissue (Figure 4a). Hematoxylin and eosin (H&E) images indicated an accumulation of cells reminding the pre-adipocytes or adipocytes phenotype (Figure 4b). Histological staining with Oil Red O demonstrated that fat tissue was increased in the TME of the SR59230A-treated mice (Figure 4c). Cytofluorimetric analysis and lipid quantification confirmed that both β-AR blockers, but predominantly SR59230A, increased the pre-adipocytes percentage within the tumor mass (Figure 4d,e).

## 3. Discussion

Among the β-AR subtypes, the selective role played by β3-AR in regulating tumor growth in a melanoma B16-F10-syngeneic model has already been confirmed through pharmacological and genetics approaches [16]. Despite the limitations in the use of SR59230A as a selective β3-AR antagonist, its action is strictly dependent on the experimental model used. In a B16-F10 *in vivo* syngeneic model, it has already been demonstrated that tumor related effects observed in the SR59230A-treated mice are similar to the results obtained by performing β3-AR siRNA silencing compared to β2-AR silencing [16]; however, a partial involvement of the other β-AR subtypes cannot be excluded.

Tumor stromal hematopoietic cells do originate from BM, and may be recruited to tumor sites from the peripheral blood through inflammatory signals [20,21,22]. HSCs have been previously indicated as a compartment of tumor stroma; moreover, it has been hypothesized that HSCs can be taught by the primary tumor to favor metastatic spreading [23]. Here, we demonstrated that pharmacological antagonism of β3-AR by using SR59230A induced a multi-lineage commitment of the HSCs recruited in the tumor microenvironment, and partially in the bone marrow niche, and that this effect impairs stromal cells sustainability to tumor progression.

Our data showed that, during the *in vivo* growth, tumor upregulates β3-AR, and its antagonism is extraordinarily effective in reducing stemness in the TME, highlighting the crucial role played by β3-ARs in favoring tumor progression. Moreover, we demonstrated that β3-AR blockade is able to induce a hematopoietic process in tumors through differentiation of different progenitor cells leading to a favorable effect to arrest cancer progression. Our results are in agreement with the previous evidence showing that β3-ARs are responsible for the recruitment of stromal cells in the TME [12]; furthermore, we detected its crucial role in regulating stromal cell differentiation. Based on their key role in drug resistance, disrupting CSCs in melanoma is considered a promising effective strategy for novel therapeutic approaches [24].

Interestingly, we have also shown that, in a syngeneic B16 melanoma tumor model, β-AR antagonists, and in particular SR59230A, could induce MSCs differentiation in adipocytes. According to these results, it has been reported that propranolol and SR59230A transdifferentiate different types of stem cells into pre-adipocytes, such as hemangioma stem cells [25], leading to a better outcome. These results reinforce the concept that reduction of the MSCs percentage in the TME, or induction of their differentiation could be an evaluable strategy to arrest melanoma growth, and that β3-AR plays a crucial role in mediating these effects.

In conclusion, our results highlight a new role of β3-ARs in controlling the differentiation of tumor stroma in the TME, hopefully opening a new strategy to counteract melanoma progression by increasing both cell death and differentiation processes.

## 4. Materials and Methods

### 4.1. Cell Culture

Murine B16-F10 melanoma cell line was obtained from the ATCC (Manassas, VA, USA). Murine B16-F10 melanoma GFP stable cell line was obtained from Creative Biogene Biotechnology (Shirley, NY, USA). Cells were maintained in the Dulbecco’s modified Eagle’s medium (DMEM) containing 10% fetal calf serum (FCS), 2 mM L-glutamine, 100 U/mL penicillin, and 100 μg/mL streptomycin (Euroclone Group, Pero, Italy) at 37 °C in 5% CO_2_. The cell line was mycoplasma-tested.

### 4.2. Mice

*In vivo* experiments and tissue collection were carried out according to the European Union (EU) guidelines for animal care procedures and the Italian legislation (DLgs 26/2014) in implementation of EU Directive 2010/63/EU. Studies were conducted under research permits issued by the University of Florence. C57BL/6 mice (male, 20–25 g, 5–6 weeks; Envigo, Milan, Italy) were used. Animals were housed in a temperature- and humidity-controlled vivarium (12 h dark/light cycle, free access to food and water, maximum 10 animals per cage). All the experiments were performed in a quiet temperature-controlled room (20–22 °C). Animals were euthanized with ketamine/xylazine (120 mg/kg and 16 mg/kg).

### 4.3. Murine B16-F10 Syngeneic Model and Treatments

B16-F10 cells were implanted in C57BL/6 recipient mice by injecting 5 × 10^5^ cells in 200 µL phosphate-buffered saline (Gibco, Gaithersburg, MD, USA) subcutaneously (s.c.) in the right flank of the mice. Mice were monitored daily. After approximately 15 days, when B16-F10 cells formed a palpable tumor, the treatment was started. The treatments were administrated twice a day with a window of 4–6 h between each treatment. DMSO (vehicle), propranolol (20 mg/kg/day, Sigma-Aldrich, St. Louis, MO, USA), and SR59230A (20 mg/kg/day, Sigma-Aldrich) were injected intraperitoneally (i.p.). Tumor growth rate was evaluated by measuring the tumor mass with a caliper, and tumor mass volume was calculated as Volume = (length × width)^2^/2. Mice were sacrificed at 10 (T10) and 20 (T20) days after starting treatment, and tumors were weighed and measured.

### 4.4. Isolation of Tumor Cells

Mouse tumor tissues were minced with scissors and incubated in C-Tubes (Miltenyi Biotec^®^, Bergisch Gladbach, Germany) with a storage tissue solution (Miltenyi Biotec^®^). Tumor samples were then homogenized using a Tumor Dissociation Kit (Miltenyi Biotec^®^) and a gentleMACS Octo Dissociator (Miltenyi Biotec^®^) with an appropriate Heaters run program. After homogenization, the samples were filtered with pre-separation filters (MACS SmartStrainers 70 μM, Miltenyi Biotec^®^) to remove cell aggregates or large particles. Lymphocytes were subsequently separated by tumor samples using anti-CD45 MicroBeads (Miltenyi Biotec^®^) and AutoMACS Separator Pro (Miltenyi Biotec^®^) according to the manufacturer’s instructions.

### 4.5. Viability Assay

B16-F10 cells isolated from mouse tumor tissues were tested for viability. Viability of tumor cells in control and treatment conditions was evaluated using Annexin V-PE (Miltenyi Biotec^®^, Bergisch Gladbach, Germany) and PI staining. The stained cells were acquired using a MACSQuant Analyzer 10 Flow Cytometer (Miltenyi Biotec^®^), and the data were processed using Flowlogic (Miltenyi Biotec^®^).

### 4.6. Extraction of Lipids from Tumors

For the extraction of lipids from tumor, the Folch method was used [26]. The tumors were homogenized with chloroform/methanol (2:1) to a final volume of 20 mL of solvent mixture per 1 g of tissue. After dispersion, the samples were kept agitated for 15 min in an orbital shaker at room temperature. Then, the homogenates were centrifuged at 5000 rpm for 10 min, and the liquid phase was recovered. The sample was washed with 4 mL of 0.9% NaCl solution, and, after vortexing for a few seconds, the mixture was centrifuged at 2000 rpm to separate the two phases. After centrifugation, the upper phase was removed, and the lower phase containing lipids was evaporated by vacuum in a rotary evaporator. After the evaporation, the amount of lipids was quantified using an analytic balance.

### 4.7. Flow Cytometry and Morphology Analysis

Cells isolated from mouse tumors were incubated and stained with various appropriately diluted (Supplementary Table S1) combinations of the following fluorochrome-conjugated antibodies: anti-CD45-VioBlue or VioGreen (130-110-802, 130-102-776), anti-CD11b-FITC or PerCP Vio700 (130-109-289), anti-Gr1-PE (130-102-426), anti-CD106-PE (130-104-712), anti-Sca1-PE Vio700 (130-106-220), anti-CD73-FITC (130-102-535), anti-CD29-APC (130-102-557), anti-CD44-VioBlue (130-116-495), anti-CD34-FITC (130-105-831), anti-CD31-PE (130-102-608), anti-CD140 (PDGFR alpha)-APC Vio 770 (130-105-119), anti-Ter119-APC (130-102-290), Lineage Cell Detection Cocktail-Biotin-APC Vio 770 (130-092-613), anti-CD49b-PE (130-102-337), anti-Ly6C-FITC (130-111-777), anti-Ly6G-APC (130-102-295), anti-CD117-PE (130-111-615), anti-CD135-APC (130-102-512), anti-CD127-APC (130-102-529), anti-CD122-FITC (130-102-481), Annexin V-PE (130-108-112), and Propidium Iodide Solution (130-093-233) (Miltenyi Biotec^®^, Bergisch Gladbach, Germany). The stained cells were acquired using a MACSQuant Analyzer 10 Flow Cytometer (Miltenyi Biotec^®^), and the data were processed using Flowlogic (Miltenyi Biotec^®^).

### 4.8. Immunohistochemistry

Sections of 4 µm thickness were cut from formalin-fixed paraffin-embedded samples of mouse tumors at T10 for Ki-67 staining, and at T10 and T20 for β2- and β3-AR staining. Immunostaining was performed according to standard procedures. Briefly, sections were deparaffinized in xylol and hydrated with grade ethanol concentrations until distilled water. Antigen retrieval was performed by immersing the slides in a thermostat bath containing 10 mM citrate buffer (pH 6.0) for 15 min at 98 °C followed by cooling for 20 min at room temperature. Endogenous peroxidase activity was blocked by treating the sections with hydrogen peroxide blocking reagent (Thermo Fisher, Rockford, IL, USA). After blocking with normal horse serum (UltraVision, Thermo Fisher), sections were incubated with the following primary antibodies: Ki-67 (1:100, rabbit polyclonal, PA5-19462, Thermo Fisher), β2-AR (ab182136, Abcam, Cambridge, UK), and β3-AR (ab94506, Abcam, Cambridge, UK). Bound antibodies were visualized using aminoethyl carbazol or 3,3′-diaminobenzidine as chromogens. Nuclei were counterstained with Mayer’s hematoxylin. Negative controls were performed by substituting the primary antibody with a non-immune serum. Quantification of the optical density (OD) in each specimen was obtained by using the Immunohistochemistry (IHC) Image Analysis Toolbox of ImageJ 1.48v software (NIH, USA).

### 4.9. Oil Red O Staining

Mouse tumor was frozen and cut in sections of 10 µm thickness. Sections were air-dried, fixed in formalin for 5 min, and washed with running tap water for 1–10 min. Then, sections were rinsed with 60% isopropanol and stained with freshly prepared Oil Red O working solution for 15 min. Sections were then rinsed with 60% isopropanol, and nuclei were counterstained with Mayer’s hematoxylin.

### 4.10. Statistics

Statistical analysis was performed using GraphPad Prism (GraphPad, San Diego, CA, USA) by one-way or two-way analysis of variance (ANOVA), followed by the Bonferroni post-hoc test. For the *in vivo* experiments, according to the previous studies on the same animal model [14], 6 mice per group were needed to guarantee a power of 80%. We performed two experiments with *n* = 3 each. Values are presented as mean ± SD. * *p* < 0.05, ** *p* < 0.01, *** *p* < 0.001, **** *p* < 0.0001, Prop compared with Veh. ### *p* < 0.001, #### *p* < 0.0001, SR compared with Veh. $ *p* < 0.05, $$ *p* < 0.01, $$$ *p* < 0.001, Prop compared with SR.

## Supplementary Materials

The following are available online at https://www.mdpi.com/1422-0067/21/4/1420/s1.
