# Supplementary material for: β3-Adrenoreceptor Blockade Induces Stem Cells Differentiation in Melanoma Microenvironment
Source: Int J Mol Sci. 2020 Feb 20;21(4):1420. doi: 10.3390/ijms21041420 (PMC7073111; doi:10.3390/ijms21041420)
Supplement: Supplementary file 1 [file ijms-21-01420-s001.pdf]

Supplementary Figure 1

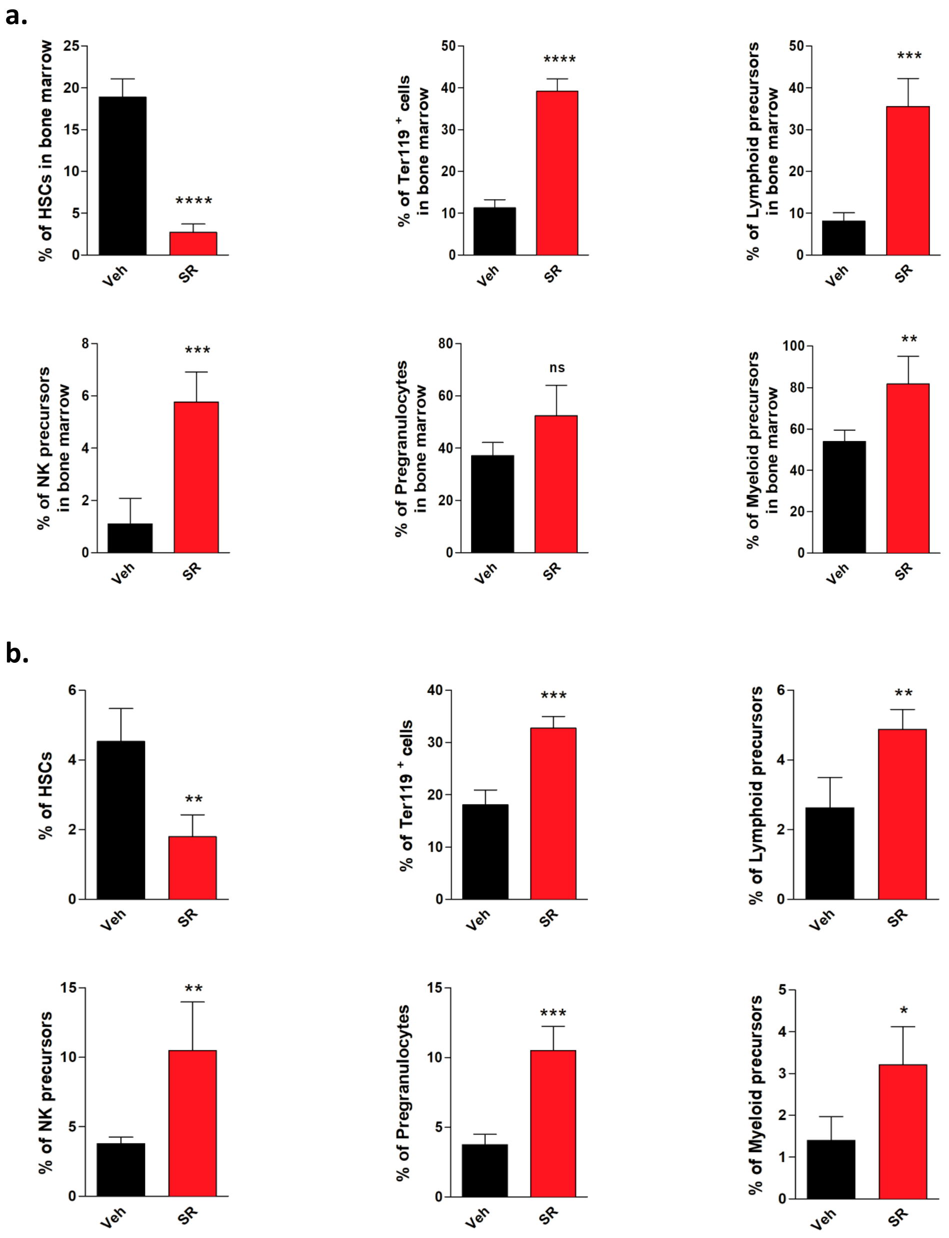

Figure S1. FACS analysis and relative quantification of HSCs (CD117^{+}, Sca1^{+} gated on Lin^{-}), Ter119 gated on CD45^{-} cells, lymphoid precursors (SCA1^{+} gated on CD117^{+}, CD127^{+}, Lin^{-}), NK precursors (NK1.1^{-} gated on CD122^{+}, Lin^{-}), pre-granulocytes (CD127^{-}, CD16/32^{+} gated on CD117^{+}, SCA1^{-}, CD34^{+}, Lin^{-}) and myeloid (SCA1^{-} gated on CD117^{-}, CD127^{+}, Lin^{-}) in **(a)** bone marrow isolated from Vehicle- and SR59230A-treated mice and in **(b)** untreated-tumor mass disrupted and cultured *ex-vivo* and then treated with Vehicle and SR59230A. Cells were then analyzed after 7 days of treatments.

pplementary Figure 2

a.

	Health	Ctrl	Veh	Prop	SR
WBC (*10.e3/uL)	3,89 ± 0,26	3,78 ± 0,28	7,29 ± 0,95	4,92 ± 0,73	4,30 ± 0,64
Neut (%)	5,3 ± 0,10	0,45 ± 0,05	0,8 ± 0,17	2,93 ± 1,49 *	1,03 ± 0,42 *
Lymph (%)	78,3 ± 1,30	48,1 ± 7,30	35,57 ± 7,92	49,07 ± 7,13	51 ± 3,59
Mono (%)	10,55 ± 1,55	46,35 ± 10,65	52,27 ± 6,35	40,23 ± 7,37	39,13 ± 4,69
Eos (%)	0,35 ± 0,05	0,07 ± 0,03	0,07 ± 0,03	0,18 ± 0,07	0,1 ± 0,03
Baso (%)	0,25 ± 0,05	0,1 ± 0,10	0,17 ± 0,12	0,18 ± 0,02	0,1 ± 0,04
LUC (%)	5,25 ± 0,15	4,9 ± 3,30	11,1 ± 1,49	7,43 ± 1,03 *	8,62 ± 1,56
RBC (*10.e6/uL)	9,33 ± 0,95	3,36 ± 0,47	5,24 ± 1	5,28 ± 1,03	4,65 ± 0,44
Hb (g/dL)	14,65 ± 0,25	5,05 ± 0,85	7,63 ± 1,43	7,67 ± 1,54	6,95 ± 0,62
Hct (%)	48 ± 4,60	20,75 ± 4,05	28,07 ± 4,37	29,03 ± 4,66	27,05 ± 2,34
MCV (fL)	51,5 ± 0,30	61,3 ± 3,50	54,43 ± 2,23	56,73 ± 2,09	58,72 ± 2,15
MCH (pg)	15,85 ± 1,35	15 ± 0,40	14,63 ± 0,15	14,55 ± 0,58	15,02 ± 0,24
MCHC (g/dL)	30,75 ± 2,35	24,5 ± 0,80	26,93 ± 1,01	25,73 ± 1,21	25,67 ± 0,69
RDW (%)	12,8 ± 0,50	19,75 ± 0,35	17,33 ± 1,39	21,25 ± 2,63	19,33 ± 1,78
HDW (g/dL)	1,785 ± 0,02	2,065 ± 0,08	2,02 ± 0,11	2,15 ± 0,17	2,12 ± 0,12
CHCM (g/dL)	25,15 ± 0,05	23,65 ± 0,55	25,43 ± 0,37	25,13 ± 0,46	24,4 ± 0,44
CH (pg)	12,9 ± 0,10	14,4 ± 0,50	13,8 ± 0,38	14,15 ± 0,47	14,2 ± 0,33
CHDW (pg)	1,65 ± 0,06	2,665 ± 0,18	2,24 ± 0,21	2,88 ± 0,43	2,59 ± 0,24
PLT (*10.e3/uL)	790 ± 9	457,5 ± 112,50	571 ± 111,38	750,67 ± 91,53	669,5 ± 125,01
MPV (fL)	5,9 ± 0,20	7,75 ± 0,15	6,17 ± 0,50	7,47 ± 0,38	8,28 ± 0,64
PDW (%)	46,9 ± 3,40	68,3 ± 10,90	41,9 ± 9,42	50,85 ± 5,18	56,72 ± 8,67
PCT (%)	0,465 ± 0,02	0,355 ± 0,10	0,36 ± 0,09	0,55 ± 0,05	0,55 ± 0,11

b.

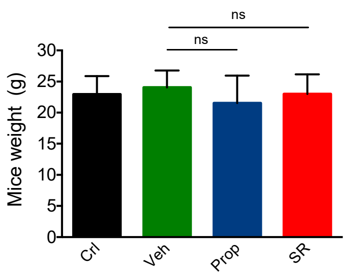

Figure S2. (**a**) Blood analysis and (**b**) mice weight measurement after 20 days of treatment.

Table S1. Fluorochrome-conjugated antibodies dilutions used in FACS analysis.

**Antibody**	**Dilution**
Anti-CD45-VioBlue (130-110-802)	1:50
Anti-CD45-VioGreen (130-102-776)	1:50
Anti-CD11b-FITC or PerCPVio700 (130-109-289)	1:50
Anti-Gr1-PE (130-102-426)	1:10
Anti-CD106-PE (130-104-712)	1:10
Anti-SCA-1-PE or Vio700 (130-106-220)	1:10
Anti-CD73-FITC (130-102-535)	1:10
Anti-CD29-APC (130-102-557)	1:10
Anti-CD44-VioBlue (130-116-495)	1:50
Anti-CD34-FITC (130-105-831)	1:50
Anti-CD31-PE (130-102-608)	1:50
Anti-CD140-APCVio770 (130-105-119)	1:50
Anti-TER119-APC (130-102-290)	1:10
Anti-Lin-APC Vio770 (130-092-613)	1:11
Anti-CD49b-PE (130-102-337)	1:50
Anti-Ly6C-FITC (130-111-777)	1:50
Anti-Ly6G-APC (130-102-295)	1:50
Anti-CD117-PE (130-111-615)	1:50
Anti-CD135-APC (130-102-512)	1:10
Anti-CD127-APC (130-102-529)	1:10
Anti-CD122-FITC (130-102-481)	1:10
Anti-AnnexinV-PE (130-108-112)	1:10
Anti-Propidium Iodide Solution (130-093-233)	1:50
